# Emergency Medicine Funding Within NIH Is the Least Concentrated of Any Specialty

**DOI:** 10.1111/acem.70316

**Published:** 2026-05-08

**Authors:** Nicholas M. Mohr, Karen I. Cyndari, Priyanka Vakkalanka, Brett Faine, Tera Shea, Manish N. Shah

**Affiliations:** ^1^ Department of Emergency Medicine University of Iowa Carver College of Medicine Iowa City Iowa USA; ^2^ Department of Anesthesia University of Iowa Carver College of Medicine Iowa City Iowa USA; ^3^ Department of Epidemiology University of Iowa College of Public Health Iowa City Iowa USA; ^4^ Department of Immunology University of Iowa Carver College of Medicine Iowa City Iowa USA; ^5^ University of Iowa College of Pharmacy Iowa City Iowa USA; ^6^ BerbeeWalsh Department of Emergency Medicine University of Wisconsin School of Medicine and Public Health Madison Wisconsin USA

Emergency medicine (EM) has been a specialty since 1979, but it ranks last among all specialties in National Institutes of Health (NIH) funding per academic faculty [1, 2]. Recognizing the diverse areas of research and the need to coordinate emergency care research across institutes, the Office of Emergency Care Research was founded in 2012 [3]. This report quantifies funding diversity by specialty, with the long‐term goal to inform strategic growth in emergency care research. As a secondary objective, we measured funding concentration by institution across specialties.

We conducted a cross‐sectional study of NIH and Agency for Healthcare Research and Quality (AHRQ)‐funded “R01” and “R01‐equivalent” grants active during calendar year 2025 (Fiscal Year = “2025,” Department Type = “Emergency Medicine,” Activity Code = “R01 Equivalents”). We extracted data from NIH RePORTER [4], then we categorized each grant by the NIH department type (specialty), organization (university), and institute/center (IC). We generated counts of the number of active grants funded by each IC within each specialty and grants awarded to each institution (university) within each specialty. We focused our data collection on counts of R01 and R01‐equivalent grants only to limit the impact of institutional awards, institute‐specific priorities for career‐level funding and career development funding, and different sizes of awards from different ICs. From those counts, we calculated the Herfindahl–Hirschman Index (HHI), which measures market concentration in macroeconomic studies [5]. The HHI index is calculated by taking the sum of the squares of the proportion of market share of all the firms in a market, with a higher value suggesting more concentrated market share (e.g., fewer firms with relatively higher market share). We calculated the HHI based on the number of grants funded by each IC within each specialty, then we calculated the HHI based on the number of grants to each institution within each specialty.

In 2025, EM had 95 funded grants across 20 ICs awarded to 32 universities. HHI of IC's across specialties ranged from 0.117 to 0.748, with EM having the least concentrated funding portfolio of any specialty (Figure 1). The National Heart, Lung, and Blood Institute (25%) had the greatest percentage of grants to EM departments, followed by the National Institute for Child Health and Human Development (13%).

**FIGURE 1:**
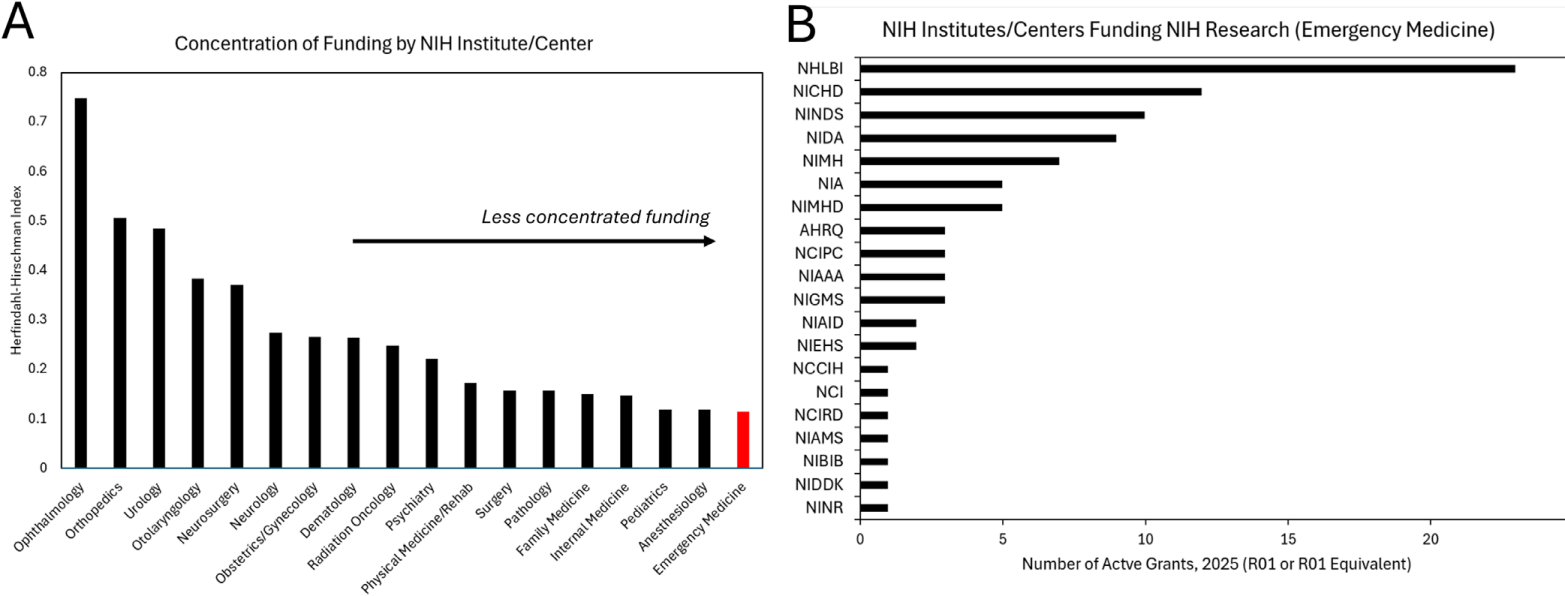
Concentration of funding by NIH Institute or Center (including AHRQ) across specialties. (A) Herfindahl–Hirschman Index (HHI) of each specialty, for R01 or R01‐equivalent grants in 2025. (B) Distribution of number of grants by NIH funding source for departments of emergency medicine.

Concentration of funding to academic institutions in EM was in the top half of all specialties, with an HHI of 0.051 (range across specialties 0.023–0.074, Figure S1). The five departments with the greatest number of NIH grants represented 40% of the total number of grants across EM. All summary data are detailed in Table S1.

Our study has several limitations. First, we evaluated only NIH and AHRQ funding because of public data accessibility, but this funding alone does not represent all funded research in EM. Second, we stratified grants by department type and institution of the single contact principal investigator. Finally, we examined only one type of career research project grant.

Among the 2030 EM Research Strategic Goals, endorsed by the Association of Academic Chairs of Emergency Medicine, the Society for Academic Emergency Medicine, the American College of Emergency Physicians, and the American Academy of Emergency Medicine, was to increase the number of NIH grant submissions and funded grants. EM is different from other specialties, though, as researchers make contributions within many related disciplines.

We believe that this lack of concentration within NIH is an advantage. Having diverse funding streams reduces the reliance on any single funding IC, which broadens the funding pool, especially during periods of budgetary instability. It also promotes diverse intellectual interests in EM—necessary given EM clinical practice. The risk is that having EM researchers represent a small portion of the portfolio of each IC may reduce the political concentration and interest in EM within any particular IC. This risk can be problematic, but it highlights the importance of advocacy within NIH such as through the ACEP‐SAEM Research Task Force and the SAEM Federal Funding Committee. The advantage of having opportunities to make scholarly contributions within diverse and emerging domains is an attractive feature, and it will inform future strategic initiatives by investigators and specialty societies to increase emergency care research across NIH.

Another of the 2030 Goals was to increase the number of EM departments with NIH‐funded research. Currently, few departments constitute most career‐level research funding. These foci of federally funded research provide unique mentorship and research infrastructure, but expanding research in only a small number of departments may limit the future scope and scale of funded researchers in EM.

In conclusion, EM enjoys a uniquely diversified NIH funding portfolio, even if the absolute number of per capita awards remains low. That funding pool is concentrated in a relatively small number of productive institutions. Future strategic initiatives will consider this feature of EM research funding to guide federal advocacy and institutional mentorship.

## Author Contributions

N.M.M. conceptualized the study, analyzed and interpreted the data, drafted the manuscript, and approved the conclusions. K.I.C., P.V., B.F., and M.N.S. interpreted the data, revised the manuscript for important intellectual content, and approved the conclusions. T.S. collected, analyzed, and interpreted the data, revised the manuscript for important intellectual content, and approved the conclusions.

## Funding

This work was supported by funding from the University of Iowa Department of Emergency Medicine.

## Conflicts of Interest

The authors declare no conflicts of interest.

## Supporting information


**Figure S1:** Concentration of funding by university or institution across specialties. (A) Herfindahl–Hirschman Index (HHI) of each specialty, for R01 or R01‐equivalent grants in 2025. (B) Distribution of number of grants by NIH funding source for departments of emergency medicine.
**Table S1:** Measures of funding concentration across specialties, stratified by NIH institute/center (IC) and by organization (university). Each specialty is defined according to the organization type in NIH RePORTER. *HHI, Herfindahl Hirschman Index*.

## Data Availability

The data that support the findings of this study are available in NIH RePORTER at https://reporter.nih.gov/. These data were derived from the following resources available in the public domain: NIH RePORTER, https://reporter.nih.gov/.
