# Supplementary material for: Emergency Medicine Funding Within NIH Is the Least Concentrated of Any Specialty
Source: Acad Emerg Med. 2026 May 8;33:e70316. doi: 10.1111/acem.70316 (PMC13155174; doi:10.1111/acem.70316)
Supplement: Supplementary file 1 — Figure S1: Concentration of funding by university or institution across specialties. (A) Herfindahl–Hirschman Index (HHI) of each specialty, for R01 or R01‐equivalent grants in 2025. (B) Distribution of number of grants by NIH funding source for departments of emergency medicine. Table S1: Measures of funding concentration across specialties, stratified by NIH institute/center (IC) and by organization (university). Each specialty is defined according to the organization type in NIH RePORTER. HHI, Herfindahl Hirschman Index. [file ACEM-33-0-s001.pdf]

Supplemental Appendix

Supplemental Figure 1. Concentration of funding by university or institution across specialties. A. Herfindahl-Hirschman Index (HHI) of each specialty, for R01 or R01-equivalent grants in 2025. **B.** Distribution of number of grants by NIH funding source for departments of emergency medicine.

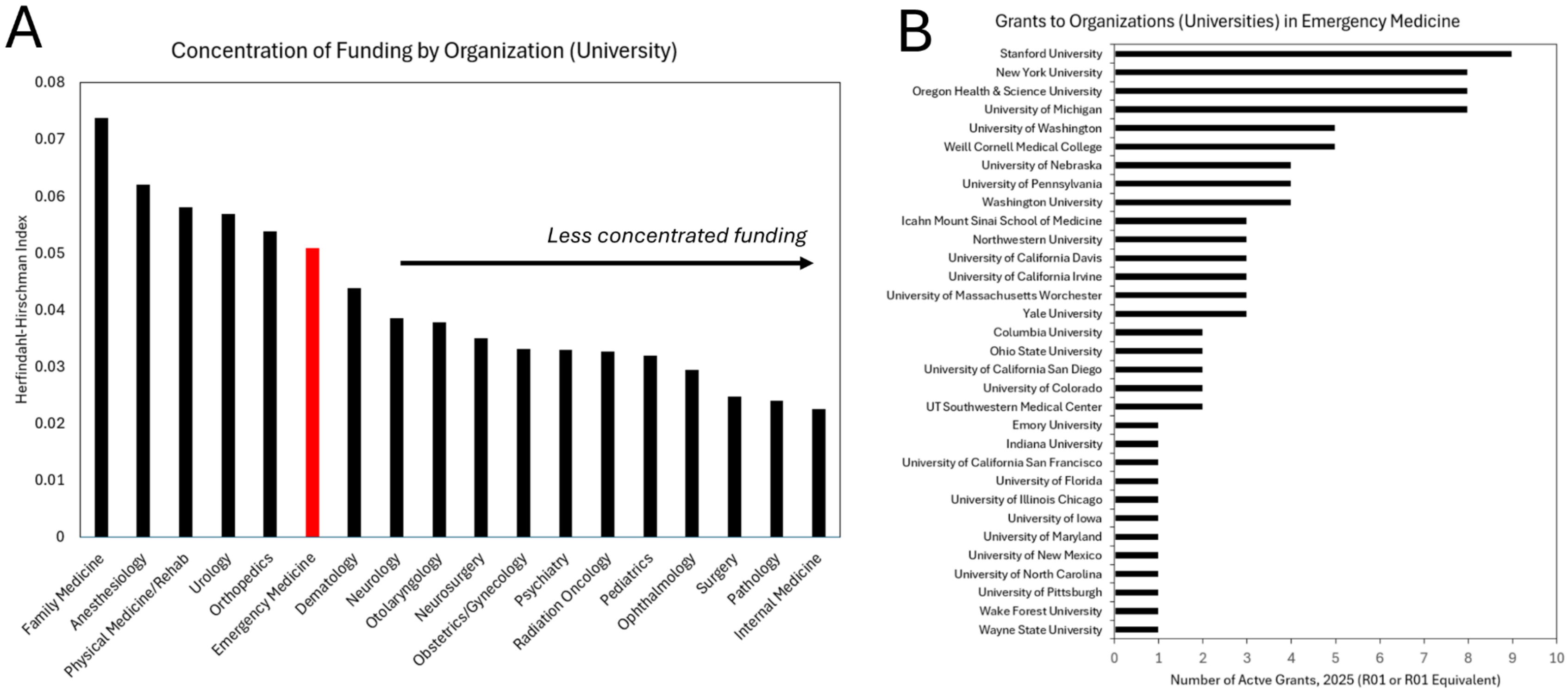

Supplemental Table 1. Measures of funding concentration across specialties, stratified by NIH institute/center (IC) and by organization (university). Each specialty is defined according to the organization type in NIH RePORTER. *HHI, Herfindahl-Hirschman Index*

	Stratified by IC			Stratified by Organization			Sum Total	
Specialty	HHI	Percent of Awards in Top 5 ICs	Number of ICs	HHI	Percent of Awards in Top 5 Organizations	Number of Organizations	Total Awards	Total Funding
Anesthesiology	0.118	70.9%	22	0.062	34.6%	46	292	$180,722,372
Dermatology	0.264	85.6%	15	0.044	32.2%	35	146	$89,421,070
Emergency Medicine	0.114	64.9%	20	0.051	40.4%	32	94	$73,068,242
Family Medicine	0.150	74.3%	15	0.074	42.2%	36	109	$64,365,076
Internal Medicine	0.147	79.1%	32	0.023	20.7%	100	4272	$2,885,894,873
Neurology	0.273	80.9%	25	0.039	31.1%	65	775	$665,712,169
Neurosurgery	0.371	88.3%	16	0.035	30.9%	49	291	$172,691,655
Obstetrics/Gynecology	0.266	80.1%	19	0.033	28.6%	58	276	$169,374,641
Ophthalmology	0.748	93.6%	14	0.030	25.5%	61	470	$244,720,150
Orthopedics	0.506	90.5%	12	0.054	43.0%	40	200	$91,769,098
Otolaryngology	0.383	86.9%	16	0.038	30.8%	46	198	$117,691,454
Pathology	0.157	74.3%	20	0.024	22.7%	82	874	$532,325,289
Pediatrics	0.118	73.0%	30	0.032	28.8%	78	1,024	$633,158,192
Physical Medicine/Rehab	0.173	85.9%	11	0.058	47.4%	34	78	$42,797,036
Psychiatry	0.222	81.9%	27	0.033	30.3%	72	1,071	$802,853,152
Radiation Oncology	0.248	83.1%	20	0.033	28.7%	67	822	$501,126,629
Surgery	0.157	78.6%	24	0.025	21.6%	67	565	$365,461,062
Urology	0.485	94.4%	10	0.057	40.4%	28	89	$50,018,331
